# Case report: A novel *ACTA1* variant in a patient with nemaline rods and increased glycogen deposition

**DOI:** 10.3389/fneur.2024.1340693

**Published:** 2024-03-04

**Authors:** Daniela Piga, Martina Rimoldi, Francesca Magri, Simona Zanotti, Laura Napoli, Michela Ripolone, Serena Pagliarani, Patrizia Ciscato, Daniele Velardo, Adele D’Amico, Enrico Bertini, Giacomo Pietro Comi, Dario Ronchi, Stefania Corti

**Affiliations:** ^1^IRCCS Fondazione Cà Granda Ospedale Maggiore Policlinico, Neurology Unit, Milan, Italy; ^2^IRCCS Fondazione Cà Granda Ospedale Maggiore Policlinico, Neuromuscular and Rare Disease Unit, Milan, Italy; ^3^IRCCS Fondazione Cà Granda Ospedale Maggiore Policlinico, Medical Genetics Unit, Milan, Italy; ^4^Unit of Neuromuscular and Neurodegenerative Disorders, Bambino Gesu’ Children’s Research Hospital, IRCCS, Rome, Italy; ^5^Department of Pathophysiology and Transplantation, Dino Ferrari Center, University of Milan, Milan, Italy

**Keywords:** *ACTA1*, skeletal muscle rods, glycogen storage, nemaline myopathy, case report

## Abstract

**Background:**

Congenital myopathies are a group of heterogeneous inherited disorders, mainly characterized by early-onset hypotonia and muscle weakness. The spectrum of clinical phenotype can be highly variable, going from very mild to severe presentations. The course also varies broadly resulting in a fatal outcome in the most severe cases but can either be benign or lead to an amelioration even in severe presentations. Muscle biopsy analysis is crucial for the identification of pathognomonic morphological features, such as core areas, nemaline bodies or rods, nuclear centralizations and congenital type 1 fibers disproportion. However, multiple abnormalities in the same muscle can be observed, making more complex the myopathological scenario.

**Case presentation:**

Here, we describe an Italian newborn presenting with severe hypotonia, respiratory insufficiency, inability to suck and swallow, requiring mechanical ventilation and gastrostomy feeding. Muscle biopsy analyzed by light microscopy showed the presence of vacuoles filled with glycogen, suggesting a metabolic myopathy, but also fuchsinophilic inclusions. Ultrastructural studies confirmed the presence of normally structured glycogen, and the presence of minirods, directing the diagnostic hypothesis toward a nemaline myopathy. An expanded Next Generation Sequencing analysis targeting congenital myopathies genes revealed the presence of a novel heterozygous c.965 T > A p. (Leu322Gln) variant in the *ACTA1* gene, which encodes the skeletal muscle alpha-actin.

**Conclusion:**

Our case expands the repertoire of molecular and pathological features observed in actinopathies. We highlight the value of ultrastructural examination to investigate the abnormalities detected at the histological level. We also emphasized the use of expanded gene panels in the molecular analysis of neuromuscular patients, especially for those ones presenting multiple bioptic alterations.

## 1 Introduction

Congenital myopathies are a group of rare congenital genetic muscle disorders, that primarily affect the structure and the function of skeletal muscles, leading to hypotonia and muscle weakness (1–3). Mutations in various genes with a crucial role in muscle development, maintenance, and contraction, have been associated with different phenotypic and histological expressions of these disorders. Because of their wide genetic and clinical heterogeneity, next-generation sequencing (NGS) has been increasingly used for their diagnosis in recent years (3–6).

While the current classification of congenital myopathies remains subject to an ongoing evaluation, because of the constant discovery of additional genes, the diagnostic algorithm still relies on muscle biopsy findings (3, 7, 8). In fact, in reference Centers for neuromuscular disorders, despite the growing tendency toward a gene-first approach in the diagnostic assessment of such complex clinical scenarios, muscle biopsy data remain crucial in orienting and/or confirming the definitive diagnoses. Among congenital myopathies, Nemaline Myopathy (NM) features the presence of nemaline bodies (NBs), that are rod-shaped structures within muscle fibers (9–11). These rods consist in protein inclusions containing Z-line proteins, and they are likely to contribute to disrupt muscle function, leading to sarcomeric dysfunction and muscle weakness (1, 12–15).

Although nemaline bodies can be considered pathognomonic features of NMs (8, 12, 16, 17), their presence does not rule out the possibility of alternative diagnoses, including acquired conditions (18). Therefore, the identification of rod-shaped structures should prompt the molecular analysis of genes associated with NM, together with those underlying other genetic forms (18).

Congenital NM has been associated with causative variants in 14 genes encoding for sarcomeric components, and for auxiliary proteins involved in the regulation of sarcomeric functions, stability, or turnover (3, 19). Deleterious variants in *ADSSL1*, *CFL2*, *KLHL40*, *KLHL41, LMOD3*, *MYO18B*, *MYPN*, *NEB* and *TNNT3* are recessively inherited, while molecular defects in *KBTBD13* display a dominant inheritance. Finally, *ACTA1*, *TPM2*, *TPM3* and *TNNT1* genes are associated with recessive or dominant NM forms. Most of NM patients present mutations in *NEB* (50% of cases) or *ACTA1* (20–30% of patients), with *ACTA1* variants representing the most common defect in patients with congenital onset or severe presentations (20–22).

*ACTA1* gene encodes for the skeletal muscle 42 kDa alpha-actin protein, whose main function is to interact with myosin during muscle contraction. Mutations in the *ACTA1* gene can disrupt the normal structure and function of the alpha-actin-1 protein, resulting in muscle weakness, hypotonia (low muscle tone), and various muscular conditions collectively referred to as “actinopathies” (23–25). Muscle biopsies of NM patients might display a rich repertoire of pathological alterations, including cores, nemaline and intranuclear bodies, actin accumulations, fiber-type disproportion, dystrophic features, and zebra bodies.

Here, we report the case of a neonatal patient, who presented clinical features of congenital myopathy and glycogen accumulations on histological and ultrastructural analyses of the muscle. The identification of nemaline bodies at electron microscopy oriented the investigation toward the discovery of a *de novo*, novel heterozygous variant in the *ACTA1* gene.

## 2 Case presentation

The patient is the second-born child to non-consanguineous healthy parents of Italian origins (Supplementary Figure 1). He was born at full term through a vaginal delivery, following a pregnancy characterized by reduced fetal movements. At birth, the baby displayed significant hypotonia and lacked spontaneous movements and breathing activity. APGAR score was 4, 6 and 8 at 1st, 5th, and 10th minute, respectively. The newborn was immediately intubated and provided with invasive mechanical ventilation. Due to his severe general conditions, the patient was promptly transferred to the Neonatal Intensive Care Unit of our hospital for further examinations and treatments. During his hospital stay, the baby required continuous mechanical ventilation. He also showed difficulties in facial expressions, sucking, swallowing, and general voluntary movements. Furthermore, a bilateral cryptorchidism was observed. When he was 54 days old, a tracheostomy and percutaneous gastrostomy were performed, following which the baby displayed a steady growth curve, with an appropriate weight gain.

Routine biochemical profiles on multiple occasions, comprehensive of Creatine phosphokinase (CPK) dosage, returned physiological results. Ophthalmological and cardiological evaluation, with the latter including electrocardiography (ECG) and echocardiography, revealed no abnormalities. Auditory brainstem response (ABR) testing showed bilateral high auditory thresholds, higher on the right side, without brainstem dysfunction; a follow-up Brainstem Auditory Evoked Response (BAER) testing was therefore recommended at 3 months of life, and this turned out to be normal.

Electromyography (EMG) showed a widespread muscle damage, suggesting a myogenic suffering, mainly involving the proximal regions of both upper al lowerlimbs.

Electroencephalogram (EEG) displayed a global alteration of the cerebral organization, with slow wave abnormalities, and absence of sleep phase transition.

**Figure 1:**
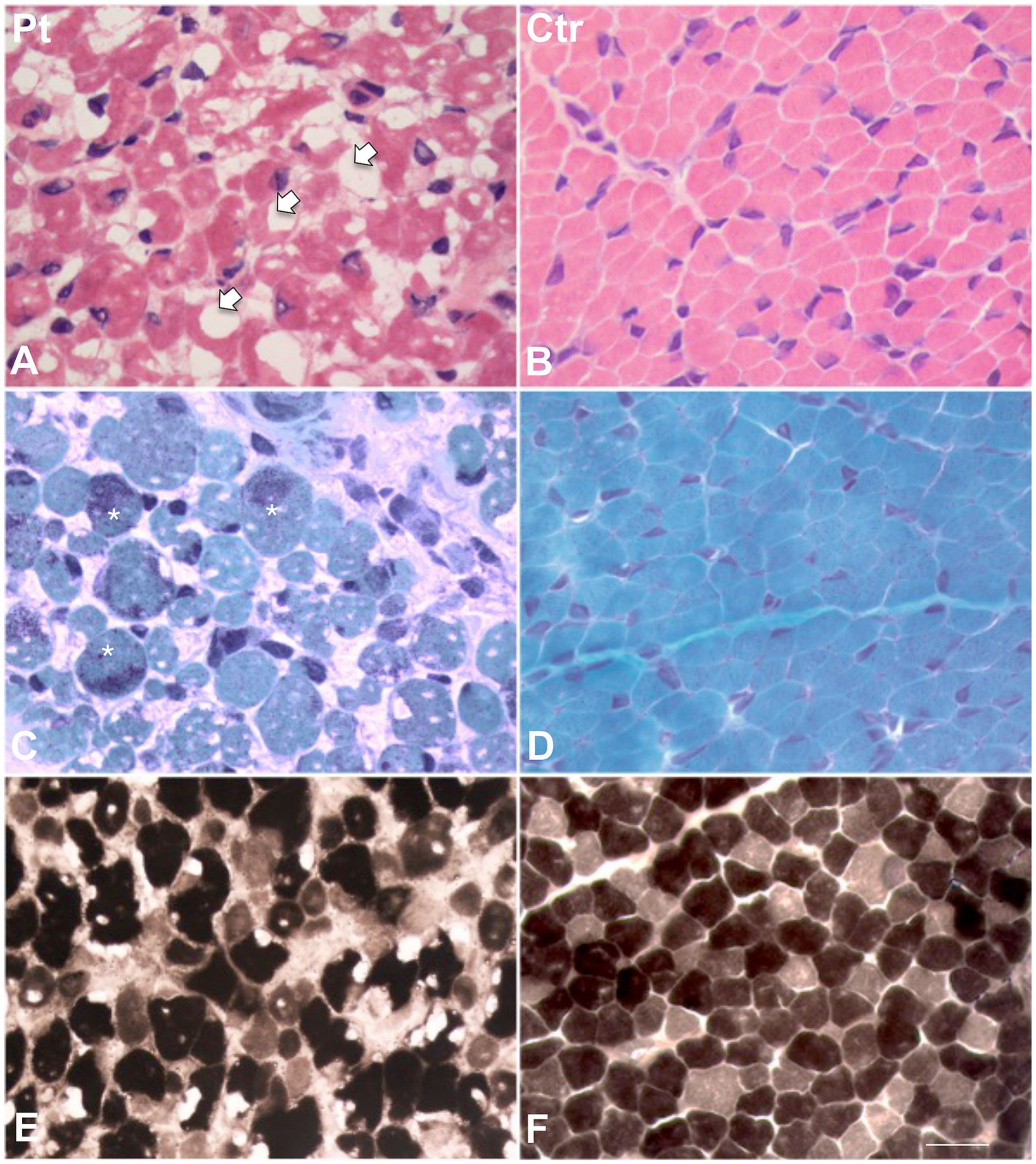
Histological findings in muscle biopsy. H&E stain in our patient **(A)** showed a great variability in fiber size. Several fibers showed the presence of cytoplasmic and subsarcolemmal optically empty vacuoles, sometimes of conspicuous size (arrows). **(B)** H&E stain in age-matched healthy control. **(C)** MGT stain in our patient showed the presence of intracytoplasmic fuchsinophilic granulations (asterisks). **(D)** MGT stain in age-matched healthy control. **(E)** ATPase pH 9.4 in our patient and **(F)** in age-matched control. Scale bar 10 μm.

**Figure 2:**
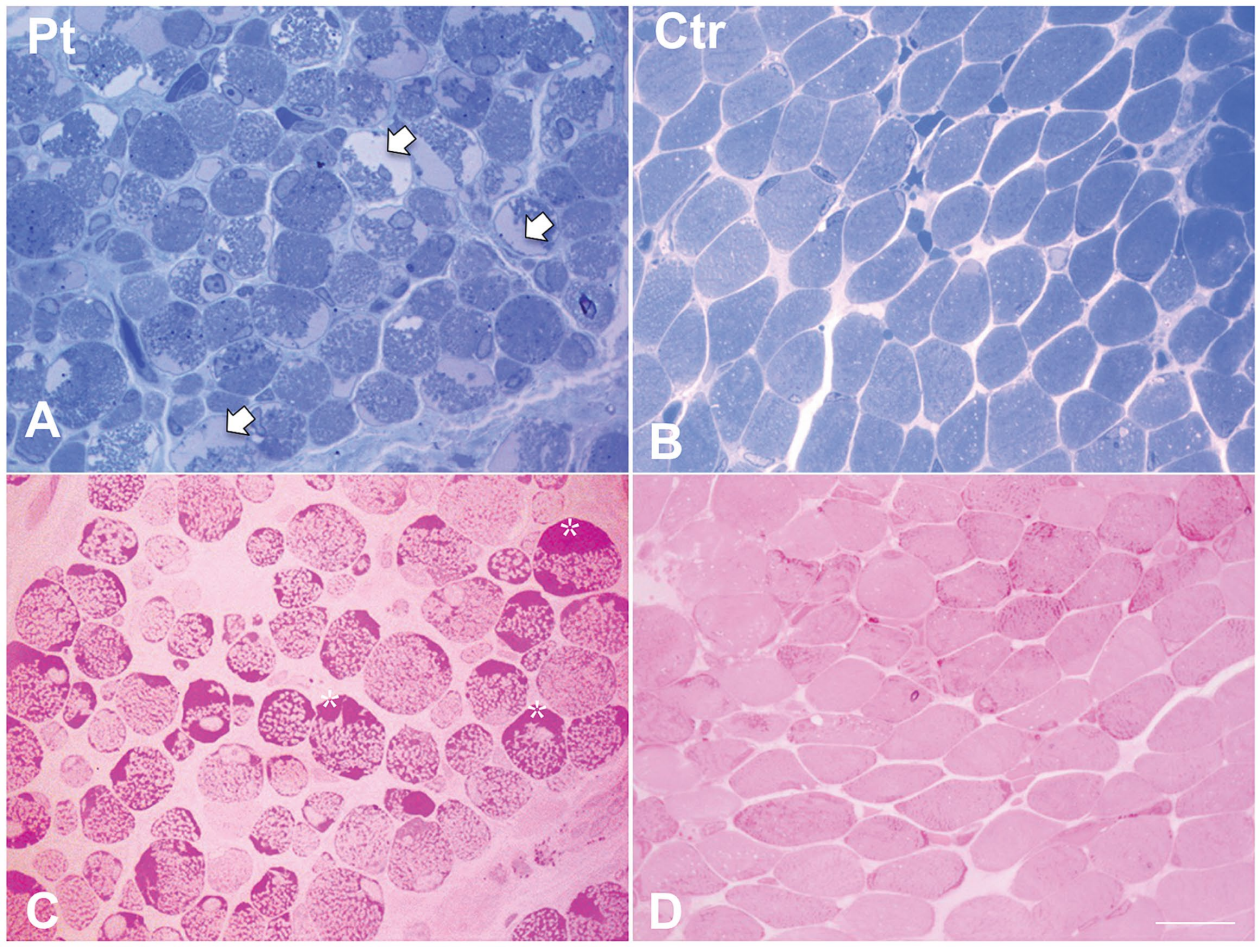
Histological findings in muscle biopsy. Toluidine blue stained semithin section in our patient **(A)** showed cytoplasmic and subsarcolemmal pale staining storage material (arrows). **(B)** Toluidine blue stained semithin section in age-matched healthy control. **(C)** PAS stain in our patient showed increased subsarcolemmal glycogen content (asterisks). **(D)** PAS stain in age-matched healthy control. Scale bar 10 μm.

**Figure 3:**
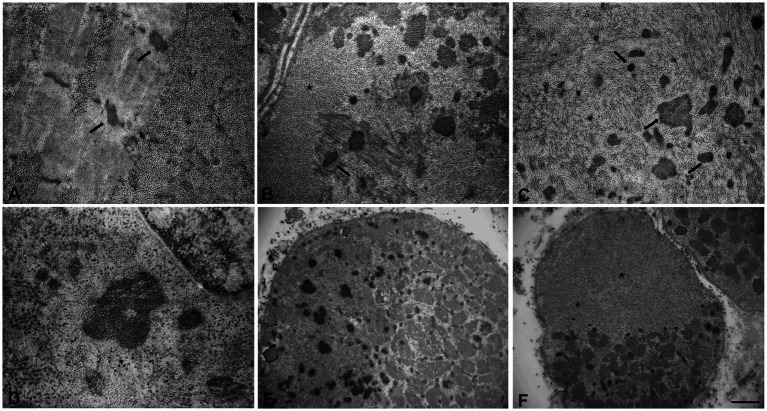
Ultrastructural analyses of the muscle sample by electron microscopy. **(A)** Glycogen granules accumulated among the myofibrils and initial thickening of Z line (arrows). **(B,C)** Large glycogen collections localized at the subsarcolemmal level and at intracytoplasmic sites, several nemaline rods of variable size and shape (arrows), myofibrillar disorganization. **(D)** High magnification of a nemaline rod and glycogen granules. **(E,F)** Fibers with areas of compartmentalization. **(E)** In the left area the muscle fiber contains several nemaline rods. **(F)** In the upper area the muscle fiber is completely replaced by glycogen. Scale bars **(A–C)**: 0.8 μm, **(D)**: 0.5 μm. **(E,F)**: 3.3 μm. **(A,B,F)** Asterisks indicate large glycogen collections.

Muscle biopsy was performed on right quadriceps, at 9 days of age. Histological analyses showed a great variability in fiber size, with a slight prevalence of type 1 fibers, and a 15% of hypotrophic fibers estimated to belong to both types of fibers. Several fibers showed the presence of cytoplasmic and subsarcolemmal optically empty vacuoles, sometimes of conspicuous size, and intracytoplasmic fuchsinophilic granulations (Figure 1). In particular, by analyzing semithin sections, we estimated the presence of rods in 14.6% of the fibers. Nuclear centralization, degenerative fibers with augmented connective tissue, fiber splittings and increased acid phosphatase staining were not observed. Analysis on semithin sections showed that the vacuoles were PAS positive, indicating an increased glycogen content (Figure 2), and suggesting a glycogen storage disease; in fact, the histopathological features resembled branching or debranching enzyme deficiency (Autosomal Recessive Glycogen Storage Disease type 4 or 3, respectively). These findings prompted the molecular analysis of the genes implied in muscle glycogen storage disorders, such as *GBE1* and *AGL*, without conclusive results. The subsequent ultrastructural analyses by electron microscopy confirmed the presence of extensive glycogen collections, leading to significant alterations in muscle architecture (Figure 3A). In addition, several cytoplasmic rods of variable size were found in some muscle fibers (Figures 3B–D). Fibers with area of compartmentalization for nemaline rods (Figure 3E) and glycogen granules (Figure 3F) were also observed.

**Figure 4:**
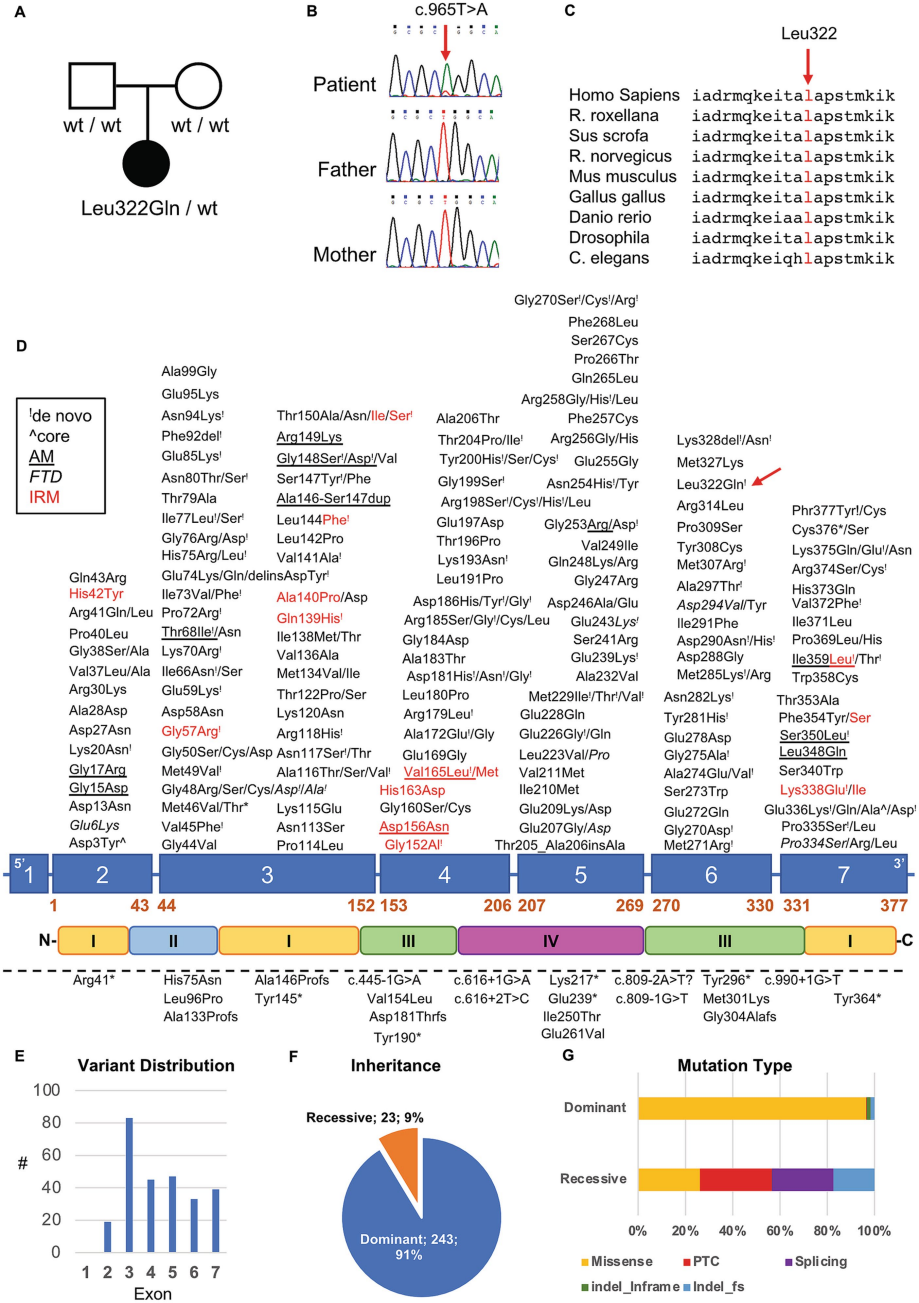
Genetic studies in our patient. **(A)** Pedigree of the family. Black symbol indicates the affected patient. **(B)** Sequence electropherograms showing the presence of the heterozygous c.965 T > A *ACTA1* variant in the patient but not in his parents suggesting *de novo* occurrence of the variant. **(C)** Evolutionary conservation of the affected residue across species. **(D)** Diagram showing the structure of *ACTA1* gene and protein. Dominant and recessive variants are indicated above and under the diagram, respectively. “!” symbol indicates a variant in which *de novo* occurrence was suspected or demonstrated; red color indicates variant associated with intranuclear rod myopathy (IRM); italic style is used for variant associated with fiber type disproportion, the underlined style is used for variants associated with muscle description of homogeneous filamentous inclusions containing actin; the “^” symbol was used to indicate variants found in patients with muscle fibers presenting core regions with no contractile material or mitochondria. Dashed lines indicate the presence of a large genomic deletion. **(E–G)** Distribution of *ACTA1* variants based on their exonic location **(E)**, inheritance pattern **(F)** and mutational types **(G)**.

To better understand such complex histological findings, additional genetic investigations were conducted. NGS sequencing analysis of a panel of genes involved in congenital myopathies revealed the presence of a novel heterozygous c.965 T > A p. (Leu322Gln) variant in the *ACTA1* gene (NM_001100.3) (Figure 4A). The absence of the variant in Patient’s parents supported the hypothesis of its *de novo* origin (Figure 4B). The variant was not found in available population databases (GnomAD MAF:0), and it is currently classified as likely pathogenic, according to the ACMG guidelines (criteria PP3-strong, PM1 and PM2). All the interrogated *in silico* prediction tools unanimously assigned a pathogenic behavior to this novel variant, which would affect an evolutionary conserved residue (Figure 4C) located in the large domain of actin, subdomain 3 (amino acids 270–337) (Figure 4D).

Eventually, the baby was discharged at 3 months and 6 days of age, with home mechanical ventilation and enteral nutrition through a gastrostomy pump. The patient is nowadays 6 years old, and he is currently nourished through a PEG tube, and yet presenting an important drooling, for which he is treated with anticholinergic drugs. He still requires continuous assisted ventilation, and his cardiological follow-up continues to show no abnormalities. He utilizes a tilting postural system for sitting, with anti-gravity support for the upper limbs. He also uses positioning braces for the lower limbs and a half-day corset, especially for seated activities. On the cognitive side, he demonstrates good abilities in attention and sensory orientation, and adequate interactive and communicative skills.

## 3 Discussion and conclusion

We describe a novel proband presenting with a severe congenital myopathy, due to a novel *ACTA1* molecular defect.

Pathogenic variants in *ACTA1* are most commonly *de novo* dominant missense mutations (90% of defects), and lead to severe NM pathology by dominant-negative effect (26) (Figures 4E–G). Rarely, *ACTA1* variants can be recessively inherited in NM patients with intrauterine onset. These variants belong to a heterogenous type of mutations that include missense substitutions, and variants predicted to alter the reading frame, or to result in truncated forms of the protein. Biallelic *ACTA1* variants might allow (24) or not (27) the expression of skeletal muscle actin, whereas the expression of the homologous cardiac actin (encoded by *ACTC1*) is generally preserved. Even if cardiac involvement is not typical of NM, dilated or hypertrophic cardiomyopathy may also be present, particularly in association with specific mutations of *ACTA1* (3, 28, 29).

Additional clinical presentations may be associated with *ACTA1* variants, and these are usually reflected by specific alterations observed at the muscle biopsy, such as: core-like areas, fiber-type disproportion, intranuclear rods, actin-containing filamentous inclusions, and zebra bodies. Some of these abnormalities are suspected to be mutation-specific, supporting the definition of a spectrum of congenital myopathies related to actin dysfunction (23).

To date, more than 250 *ACTA1* pathogenic variants have been reported as pathogenic, spanning all the coding exons of the gene, hence affecting the entire 3D structure of the encoded actin protein (20, 24, 30). Several variants associated with congenital myopathy are known to be located in close proximity to the proposed actin–tropomyosin contact sites (31). The novel amino acid change described in this report would also impinge in the same region. Amino-acidic changes have been demonstrated to increase the aggregation of actin, potentially leading to nemaline rods (26). Nevertheless, a prediction of the pathogenic effect for *ACTA1* variants is complicated by the dynamic nature of the protein (limiting the efficacy of structural modeling), and by the presence of several actin binding proteins, that can influence several aspects of actin’s function and turnover, through allosteric interactions. The partial knowledge of these aspects hampers genotype–phenotype correlations and the design of innovative therapies.

Clinically, the *ACTA1*-related NM often exhibits severe congenital forms leading to respiratory failure with death within the first year of life, though mild or childhood onset forms have also been reported (20, 32).

In early-onset NM linked to *ACTA1* variants, affected newborns present with floppy appearance due to neonatal hypotonia and severe congenital muscle weakness impairing the achievement of developmental milestones (2, 20). The weakness also affects the respiratory muscles, leading to breathing difficulties within the first hours of life. In many cases, patients require tracheostomy for mechanical ventilation. In addition to this, weakness can impair swallowing and feeding, contributing to poor weight gain. Consequently, some affected patients necessitate tube feeding and gastrostomy.

Joint contractures, facial weakness with high-arched palate, fine and gross motor delays, skeletal deformities, such as clubfeet, pectus excavatum or scoliosis, and muscle atrophy are not so uncommon (3, 18, 20, 32). Antenatal presentation, with reduced fetal movements and quantitative alteration of the amniotic fluid, have also been reported (20).

Inter- and intrafamilial clinical variability has been described in individuals with NM due to *ACTA1* pathogenic variants (30). Despite the abovementioned phenotypic variability, a correlation seems to emerge between the position of the mutation and its clinical and histological presentations (31–33). Interestingly, in a recent cohort study (20), patients harboring biallelic null *ACTA1* variants showed an increased life expectancy; this outcome was explained by the higher expression levels of cardiac alpha-actin in muscle samples. Cardiac alpha-actin is the main alpha-actin form in skeletal muscle during embryonic development, and it is then replaced by skeletal muscle alpha-actin around birth (20). The total absence of skeletal muscle alpha-actin in the skeletal muscles of these NM patients (due to the presence of biallelic null *ACTA1* variants), probably led to an increased expression of cardiac alpha-actin after birth, which might have contributed to a less severe disease course.

Our patient presented a typical congenital form of NM due to *ACTA1* mutations, with severe and generalized skeletal muscle weakness, hypotonia and lack of spontaneous movements. Disease onset was antenatal, since reduced fetal movements were reported during pregnancy. At birth, the patient immediately required assisted ventilation and nutrition. He also presented bilateral cryptorchidism, which is often reported in other forms of congenital myopathies (3). Repeated cardiological assessments excluded structural and functional anomalies. Nowadays our patient, who is 6-year-old, is still ventilated and fed with gastrostomy but no cardiological impairment has been so far observed. He displays adequate interactive and communicative skills.

A preliminary histological assessment disclosed a significant accumulation of PAS-positive material, while intracytoplasmic fuchsinophilic granulations were observed only in less than 10% of the fibers. This observation initially led to the suspicion of a glycogen storage disorder (GSD) and, consequently, to perform specific analyses on *GBE1* and *AGL*, ruling out the presence of pathogenic variants in both genes. These negative results prompted us to focus more accurately on the fuchsinophilic granulations, that were therefore studied also at the ultrastructural level. This further analysis confirmed increased glycogen content, but also revealed the presence of nemaline rods within fibers, often scattered in the glycogen. In addition, we observed a disorganized sarcomeric architecture with myofibrillar disarray, a finding which is more frequently seen in patients with more severe clinical presentations. Indeed, the degree of severity of sarcomeric disorganization is acknowledged as a more reliable prognostic marker compared to the percentage of positive fibers for nemaline bodies (34). In fact, the complete or partial absence of the typical nemaline rods is not uncommon, especially in newborns, since the detection of these rod-shaped structures depends on the time of sampling as well as on the muscle analyzed. Moreover, in neonatal cases, the physiological small dimension of muscle fibers, further reduced by atrophy or hypotrophy, can complicate the observation (20, 35).

Therefore, the employment of ultrastructural analyses is a helpful approach for the detection of these pathological structures.

Interestingly, accumulations of glycogen were also observed in the muscle biopsies from other patients carrying *ACTA1* mutations (10, 36, 37). However, there are conflicting opinions on whether glycogen storage might be a consistent feature of the *ACTA1*-related NM. Notably, glycogen accumulation was observed in the muscles of 11 NM patients with pathogenic variants in *ACTA1*, but ultrastructural confirmation was obtained in only three of them (35). In contrast, a recent in-depth examination of muscle biopsies from other 10 subjects with congenital or pediatric clinical onset did not disclose this finding (20). Moreover, a correlation between glycogen accumulation and the site or type of molecular defects is also missing. Glycogen accumulation is not typically observed in other forms of congenital myopathy, including those linked to the most frequent *NEB* mutations (34). Furthermore, *ACTA1* mouse models, that nicely recapitulate several aspects of human myopathology, also fail to show increased glycogen content (38, 39).

A defect in energy utilization has been hypothesized as possible mechanism underlying such glycogen accumulations in *ACTA1*-mutated muscles (40). In support of this hypothesis, a downregulation of the genes involved in glucose and glycogen metabolism was observed in muscle biopsies collected from 12 NM patients, and, interestingly, one of them was *ACTA1* mutated (40).

The impaired breakdown of glycogen could be the consequence of altered activity of glycogen phosphorylase, the main contributor of cytosolic glycogen lysis. This enzyme was found to interact with structural muscle proteins, including alpha actinin and F-actin (41). Alpha-actinin deficiency has been associated with increased glycogen content in a mouse model (42). Finally, we cannot exclude that pathological changes acting in the muscle of patients harboring *ACTA1* pathogenic variants could influence phosphorylase regulation by post-translational or epigenetic mechanisms (43).

To date, there is no availability of any specific pharmacological treatments for NM, and this is mainly due to the complexity of the clinical and histological presentations of the disease, which makes the definition of a phenotype–genotype correlation and the development of targeted therapies utterly challenging.

The possibility to predict the clinical course of NM based on genotype could enhance the clinical management and thereby the outcomes of the affected patients. Appropriate medical management, including physical respiratory and nutritional support, where needed, can play a pivotal role in improving the quality of life for individuals with this condition (33, 44, 45). Muscle mass augmenting exercise seem to be beneficial for patients with certain *ACTA1* mutations (10, 46). Several studies of *ACTA1*-NM mouse models demonstrated the ameliorating effects on the clinical course of specific factors inducing fiber hypertrophy (i.e., myostatin inhibitors), as well as of dietary tyrosine supplementation, hence suggesting potential targets for *ACTA1* disease therapies (47, 48). In a *ACTA1*-NM mouse model harboring the His40Tyr variant, Lindqvist and colleagues (49) performed intramuscular injections of recombinant adeno-associated viral vectors with a myosin transgene able to facilitate muscle contraction. When present, the transgene leads to restoration of the intrinsic force-generating capacity and avoids fiber atrophy.

In addition, other studies evaluated the therapeutic effects of the use of small molecules modulating calcium release from troponin C. These substrates are able to sensitize the contractile apparatus to Ca2+, subsequently activating troponin, with the result of improving muscle contraction in neuromuscular disorders, including *ACTA1*-related disease (23, 46, 50). Finally, Sztal and colleagues revealed a less severe manifestation of the *ACTA1*-NM due to an increase in transcriptional activity of an actin paralogue in a zebrafish disease model (51).

Our report highlights the clinical utility of electron microscopy to drive and support molecular testing. Nowadays, the diagnosis of inherited neuromuscular disorders usually relies on NGS protocols that are performed soon after clinical and instrumental examinations, bypassing the need for invasive muscle biopsy procedures. However, molecular testing and muscle biopsy are not mutually exclusive. For example, in neonatal and infantile-onset congenital hypotonia, muscle biopsy can often lead to a precise diagnosis alone or facilitate the orientation of the genetic testing. In the pediatric population of neuromuscular patients, the diagnostic yield was higher when genetic testing was matched with muscle biopsy findings (52). Congenital myopathies in particular show the highest degree of agreement between muscle biopsies findings and genetic results (53).

## Data availability statement

The raw data supporting the conclusions of this article will be made available by the authors, without undue reservation.

## Ethics statement

The studies involving humans were approved by “Comitato Etico Milano Area 2 Fondazione IRCCS Ca’ Granda Ospedale Maggiore Policlinico” (Milan, Italy). The studies were conducted in accordance with the local legislation and institutional requirements. Written informed consent for participation in this study was provided by the participants’ legal guardians/next of kin. Written informed consent was obtained from the minor(s)’ legal guardian/next of kin for the publication of any potentially identifiable images or data included in this article.

## Author contributions

DP: Conceptualization, Data curation, Writing – original draft. MaR: Conceptualization, Data curation, Writing – original draft. FM: Writing – review & editing. SZ: Writing – review & editing. LN: Writing – review & editing. MiR: Writing – review & editing. SP: Writing – review & editing. PC: Writing – review & editing. DV: Writing – review & editing. AD’A: Writing – review & editing. EB: Writing – review & editing. GC: Writing – review & editing. DR: Conceptualization, Data curation, Investigation, Writing – original draft, Writing – review & editing. SC: Writing – review & editing.

## References

[1] Goebel HH Anderson JR Hübner C Oexle K Warlo I. Congenital myopathy with excess of thin myofilaments. Neuromuscul Disord. (1997) 7:160–8. doi: 10.1016/S0960-8966(97)00441-0, PMID: 9185179

[2] Colombo I Scoto M Manzur AY Robb SA Maggi L Gowda V. Congenital myopathies: natural history of a large pediatric cohort. Neurol Int. (2015) 84:28–35. doi: 10.1212/WNL.0000000000001110, PMID: 25428687 PMC4336094

[3] Cassandrini D Trovato R Rubegni A Lenzi S Fiorillo C Baldacci J. Congenital myopathies: clinical phenotypes and new diagnostic tools. Italian J Pediat. (2017) 43:101. doi: 10.1186/s13052-017-0419-z, PMID: 29141652 PMC5688763

[4] Boycott KM Vanstone MR Bulman DE MacKenzie AE. Rare-disease genetics in the era of next-generation sequencing: discovery to translation. Nat Rev Genet. (2013) 14:681–91. doi: 10.1038/nrg3555, PMID: 23999272

[5] Bowdin SC. The clinical utility of next-generation sequencing in the neonatal intensive care unit. CMAJ. (2016) 188:786–7. doi: 10.1503/cmaj.160490, PMID: 27241783 PMC4978569

[6] François-Heude MC Walther-Louvier U Espil-Taris C Beze-Beyrie P Rivier F Baudou E. Evaluating next-generation sequencing in neuromuscular diseases with neonatal respiratory distress. Eur J Paediat Neurol. (2021) 31:78–87. doi: 10.1016/j.ejpn.2021.01.011 33667896

[7] Tubridy N Fontaine B Eymard B. Congenital myopathies and congenital muscular dystrophies. Curr Opin Neurol. (2001) 14:575–82. doi: 10.1097/00019052-200110000-00005 11562568

[8] North KN Wang CH Clarke N Jungbluth H Vainzof M Dowling JJ. Approach to the diagnosis of congenital myopathies. Neuromuscul Disord. (2014) 24:97–116. doi: 10.1016/j.nmd.2013.11.003, PMID: 24456932 PMC5257342

[9] Buxmann H Schlösser R Schlote W Sewell A Nowak KJ Laing NG. Congenital nemaline myopathy due to ACTA1-gene mutation and carnitine insufficiency: a case report. Neuropediatrics. (2001) 32:267–70. doi: 10.1055/s-2001-19122, PMID: 11748499

[10] Ilkovski B Cooper ST Nowak K Ryan MM Yang N Schnell C. Nemaline myopathy caused by mutations in the muscle α-skeletal-actin gene. Am J Hum Genet. (2001) 68:1333–43. doi: 10.1086/320605 11333380 PMC1226120

[11] Sparrow JC Nowak KJ Durling HJ Beggs AH Wallgren-Pettersson C Romero N. Muscle disease caused by mutations in the skeletal muscle alpha-actin gene (ACTA1). Neuromuscular Disord. (2003) 13:519–31. doi: 10.1016/S0960-8966(03)00101-9 12921789

[12] Barohn RJ Jackson CE Kagan-Hallet KS. Neonatal nemaline myopathy with abundant intranuclear rods. Neuromuscul Disord. (1994) 4:513–20. doi: 10.1016/0960-8966(94)90092-2, PMID: 7881297

[13] Goebel HH Warlo I. Gene-related protein surplus myopathies. Mol Genet Metab. (2000) 71:267–75. doi: 10.1006/mgme.2000.3064 11001821

[14] Corbett MA Robinson CS Dunglison GF Yang N Joya JE Stewart AW. A mutation in α-tropomyosinslow affects muscle strength, maturation and hypertrophy in a mouse model for nemaline myopathy. Hum Mol Genet. (2001) 10:317–28. doi: 10.1093/hmg/10.4.317, PMID: 11157795

[15] Joureau B de Winter JM Conijn S Bogaards SJP Kovacevic I Kalganov A. Dysfunctional sarcomere contractility contributes to muscle weakness in ACTA1-related nemaline myopathy (NEM3). Ann Neurol. (2018) 83:269–82. doi: 10.1002/ana.25144, PMID: 29328520 PMC5821533

[16] Schröder JM Durling H Laing N. Actin myopathy with nemaline bodies, intranuclear rods, and a heterozygous mutation in ACTA1 (Asp154Asn). Acta Neuropathol. (2004) 108:250–6. doi: 10.1007/s00401-004-0888-1 15221331

[17] Hutchinson DO Charlton A Laing NG Ilkovski B North KN. Autosomal dominant nemaline myopathy with intranuclear rods due to mutation of the skeletal muscle ACTA1 gene: clinical and pathological variability within a kindred. Neuromuscular Disord. (2006) 16:113–21. doi: 10.1016/j.nmd.2005.11.004, PMID: 16427282

[18] North KN Ryan MM. Nemaline Myopathy – RETIRED CHAPTER, FOR HISTORICAL REFERENCE ONLY In: Adam MP Mirzaa GM Pagon RA Wallace SE Bean LJ Gripp KW, editors. GeneReviews^®^. Seattle (WA): University of Washington, Seattle (1993)

[19] Clarkson E Costa CF Machesky LM. Congenital myopathies: diseases of the actin cytoskeleton. J Pathol. (2004) 204:407–17. doi: 10.1002/path.1648 15495263

[20] Labasse C Brochier G Taratuto AL Cadot B Rendu J Monges S. Severe ACTA1-related nemaline myopathy: intranuclear rods, cytoplasmic bodies, and enlarged perinuclear space as characteristic pathological features on muscle biopsies. Acta Neuropathol Commun. (2022) 10:101. doi: 10.1186/s40478-022-01400-0, PMID: 35810298 PMC9271256

[21] Laitila JM McNamara EL Wingate CD Goullee H Ross JA Taylor RL. Nebulin nemaline myopathy recapitulated in a compound heterozygous mouse model with both a missense and a nonsense mutation in Neb. Acta Neuropathol Commun. (2020) 8:18. doi: 10.1186/s40478-020-0893-1, PMID: 32066503 PMC7027239

[22] Yin X Pu C Wang Z Li K Wang H. Clinico-pathological features and mutational spectrum of 16 nemaline myopathy patients from a Chinese neuromuscular center. Acta Neurol Belg. (2022) 122:631–9. doi: 10.1007/s13760-020-01542-9, PMID: 33742414 PMC9170660

[23] Nowak KJ Ravenscroft G Laing NG. Skeletal muscle α-actin diseases (actinopathies): pathology and mechanisms. Acta Neuropathol. (2013) 125:19–32. doi: 10.1007/s00401-012-1019-z, PMID: 22825594

[24] O’Grady GL Best HA Oates EC Kaur S Charlton A Brammah S. Recessive ACTA1 variant causes congenital muscular dystrophy with rigid spine. Eur J Hum Genet. (2015) 23:883–6. doi: 10.1038/ejhg.2014.169, PMID: 25182138 PMC4795062

[25] Zukosky K Meilleur K Traynor BJ Dastgir J Medne L Devoto M. Association of a novel ACTA1 mutation with a dominant progressive scapuloperoneal myopathy in an extended family. JAMA Neurol. (2015) 72:689–98. doi: 10.1001/jamaneurol.2015.37, PMID: 25938801 PMC4461456

[26] Ilkovski B. Evidence for a dominant-negative effect in ACTA1 nemaline myopathy caused by abnormal folding, aggregation and altered polymerization of mutant actin isoforms. Hum Mol Genet. (2004) 13:1727–43. doi: 10.1093/hmg/ddh185 PMID: 15198992

[27] Nowak KJ Sewry CA Navarro C Squier W Reina C Ricoy JR. Nemaline myopathy caused by absence of α-skeletal muscle actin. Ann Neurol. (2007) 61:175–84. doi: 10.1002/ana.21035 PMID: 17187373

[28] Kaindl A Ruschendorf F Krause S Goebel H Koehler K Becker C. Missense mutations of ACTA1 cause dominant congenital myopathy with cores. J Med Genet. (2004) 41:842–8. doi: 10.1136/jmg.2004.020271, PMID: 15520409 PMC1735626

[29] D’Amico A Graziano C Pacileo G Petrini S Nowak KJ Boldrini R. Fatal hypertrophic cardiomyopathy and nemaline myopathy associated with ACTA1 K336E mutation. Neuromuscul Disord. (2006) 16:548–52. doi: 10.1016/j.nmd.2006.07.005 16945537

[30] Lehtokari VL Gardberg M Pelin K Wallgren-Pettersson C. Clinically variable nemaline myopathy in a three-generation family caused by mutation of the skeletal muscle alpha-actin gene. Neuromuscular Disord. (2018) 28:323–6. doi: 10.1016/j.nmd.2017.12.009, PMID: 29433794

[31] Feng JJ Marston S. Genotype–phenotype correlations in ACTA1 mutations that cause congenital myopathies. Neuromuscular Disord. (2009) 19:6–16. doi: 10.1016/j.nmd.2008.09.005, PMID: 18976909

[32] Moreno CAM Abath Neto O Donkervoort S Hu Y Reed UC Oliveira ASB. Clinical and histologic findings in ACTA1-related nemaline myopathy: case series and review of the literature. Pediatr Neurol. (2017) 75:11–6. doi: 10.1016/j.pediatrneurol.2017.04.002 28780987

[33] Wallgren-Pettersson C Bushby K Mellies U Simonds A. 117th ENMC workshop: ventilatory support in congenital neuromuscular disorders — congenital myopathies, congenital muscular dystrophies, congenital myotonic dystrophy and SMA (II) 4–6 April 2003, Naarden, the Netherlands. Neuromuscular Disord. (2004) 14:56–69. doi: 10.1016/j.nmd.2003.09.003, PMID: 14659414

[34] Malfatti E Romero NB. Nemaline myopathies: state of the art. Revue Neurol. (2016) 172:614–9. doi: 10.1016/j.neurol.2016.08.004, PMID: 27659899

[35] Ryan MM Ilkovski B Strickland CD Schnell C Sanoudou D Midgett C. Clinical course correlates poorly with muscle pathology in nemaline myopathy. Neurol Int. (2003) 60:665–73. doi: 10.1212/01.WNL.0000046585.81304.BC, PMID: 12601110

[36] Lim DS Roberts R Marian AJ. Expression profiling of cardiac genes in human hypertrophic cardiomyopathy: insight into the pathogenesis of phenotypes. J Am Coll Cardiol. (2001) 38:1175–80. doi: 10.1016/S0735-1097(01)01509-1, PMID: 11583900 PMC2776821

[37] Nguyen MAT Hardeman EC. Mouse models for thin filament disease In: Laing NG, editor. The sarcomere and skeletal muscle disease. New York, NY: Springer (2008). 66–77. 10.1007/978-0-387-84847-1_6 19181094

[38] Crawford K Flick R Close L Shelly D Paul R Bove K. Mice lacking skeletal muscle actin show reduced muscle strength and growth deficits and die during the neonatal period. Mol Cell Biol. (2002) 22:5887–96. doi: 10.1128/MCB.22.16.5887-5896.2002, PMID: 12138199 PMC133984

[39] Ravenscroft G Jackaman C Bringans S Papadimitriou JM Griffiths LM McNamara E. Mouse models of dominant ACTA1 disease recapitulate human disease and provide insight into therapies. Brain. (2011) 134:1101–15. doi: 10.1093/brain/awr004, PMID: 21303860

[40] Sanoudou D Haslett JN Kho AT Guo S Gazda HT Greenberg SA. Expression profiling reveals altered satellite cell numbers and glycolytic enzyme transcription in nemaline myopathy muscle. Proc Natl Acad Sci U S A. (2003) 100:4666–71. doi: 10.1073/pnas.0330960100, PMID: 12677001 PMC153613

[41] Chowrashi P Mittal B Sanger JM Sanger JW. Amorphin is phosphorylase; phosphorylase is an alpha-actinin-binding protein. Cell Motility. (2002) 53:125–35. doi: 10.1002/cm.10059, PMID: 12211109

[42] Quinlan KGR Seto JT Turner N Vandebrouck A Floetenmeyer M Macarthur DG. α-Actinin-3 deficiency results in reduced glycogen phosphorylase activity and altered calcium handling in skeletal muscle. Hum Mol Genet. (2010) 19:1335–46. doi: 10.1093/hmg/ddq010 20089531

[43] Migocka-Patrzałek M Elias M. Muscle glycogen phosphorylase and its functional partners in health and disease. Cells. (2021) 10:883. doi: 10.3390/cells10040883, PMID: 33924466 PMC8070155

[44] Wallgren-Pettersson C Laing NG. 109th ENMC international workshop: 5th workshop on nemaline myopathy, 11th–13th October 2002, Naarden, the Netherlands. Neuromuscular Disord. (2003) 13:501–7. doi: 10.1016/S0960-8966(03)00007-5 12899878

[45] Wang CH Dowling JJ North K Schroth MK Sejersen T Shapiro F. Consensus statement on standard of care for congenital myopathies. J Child Neurol. (2012) 27:363–82. doi: 10.1177/0883073812436605, PMID: 22431881 PMC5234865

[46] Nguyen MAT Joya JE Kee AJ Domazetovska A Yang N Hook JW. Hypertrophy and dietary tyrosine ameliorate the phenotypes of a mouse model of severe nemaline myopathy. Brain. (2011) 134:3516–29. doi: 10.1093/brain/awr274, PMID: 22067542

[47] Tinklenberg J Meng H Yang L Liu F Hoffmann RG Dasgupta M. Treatment with ActRIIB-mFc produces myofiber growth and improves lifespan in the Acta1 H40Y murine model of nemaline myopathy. Am J Pathol. (2016) 186:1568–81. doi: 10.1016/j.ajpath.2016.02.008, PMID: 27102768 PMC4901141

[48] Tinklenberg JA Siebers EM Beatka MJ Meng H Yang L Zhang Z. Myostatin inhibition using mRK35 produces skeletal muscle growth and tubular aggregate formation in wild type and TgACTA1D286G nemaline myopathy mice. Hum Mol Genet. (2018) 27:638–48. doi: 10.1093/hmg/ddx431, PMID: 29293963 PMC5886278

[49] Lindqvist J Levy Y Pati-Alam A Hardeman EC Gregorevic P Ochala J. Modulating myosin restores muscle function in a mouse model of nemaline myopathy. Ann Neurol. (2016) 79:717–25. doi: 10.1002/ana.24619, PMID: 26891371 PMC4950341

[50] Russell AJ Hartman JJ Hinken AC Muci AR Kawas R Driscoll L. Activation of fast skeletal muscle troponin as a potential therapeutic approach for treating neuromuscular diseases. Nat Med. (2012) 18:452–5. doi: 10.1038/nm.2618 22344294 PMC3296825

[51] Sztal TE McKaige EA Williams C Ruparelia AA Bryson-Richardson RJ. Genetic compensation triggered by actin mutation prevents the muscle damage caused by loss of actin protein. PLoS Genet. (2018) 14:e1007212. doi: 10.1371/journal.pgen.1007212, PMID: 29420541 PMC5821405

[52] Yang K Iannaccone S Burkhalter LS Reisch J Cai C Schindel D. Role of nerve and muscle biopsies in pediatric patients in the era of genetic testing. J Surg Res. (2019) 243:27–32. doi: 10.1016/j.jss.2019.04.085, PMID: 31151034

[53] Veneruso M Fiorillo C Broda P Baratto S Traverso M Donati A. The role of muscle biopsy in diagnostic process of infant hypotonia: from clinical classification to the genetic outcome. Front Neurol. (2021) 12:735488. doi: 10.3389/fneur.2021.735488, PMID: 34675869 PMC8523832

