# Supplementary figures and images for: Case report: A novel ACTA1 variant in a patient with nemaline rods and increased glycogen deposition
Source: Front Neurol. 2024 Mar 4;15:1340693. doi: 10.3389/fneur.2024.1340693 (PMC10944937; doi:10.3389/fneur.2024.1340693)

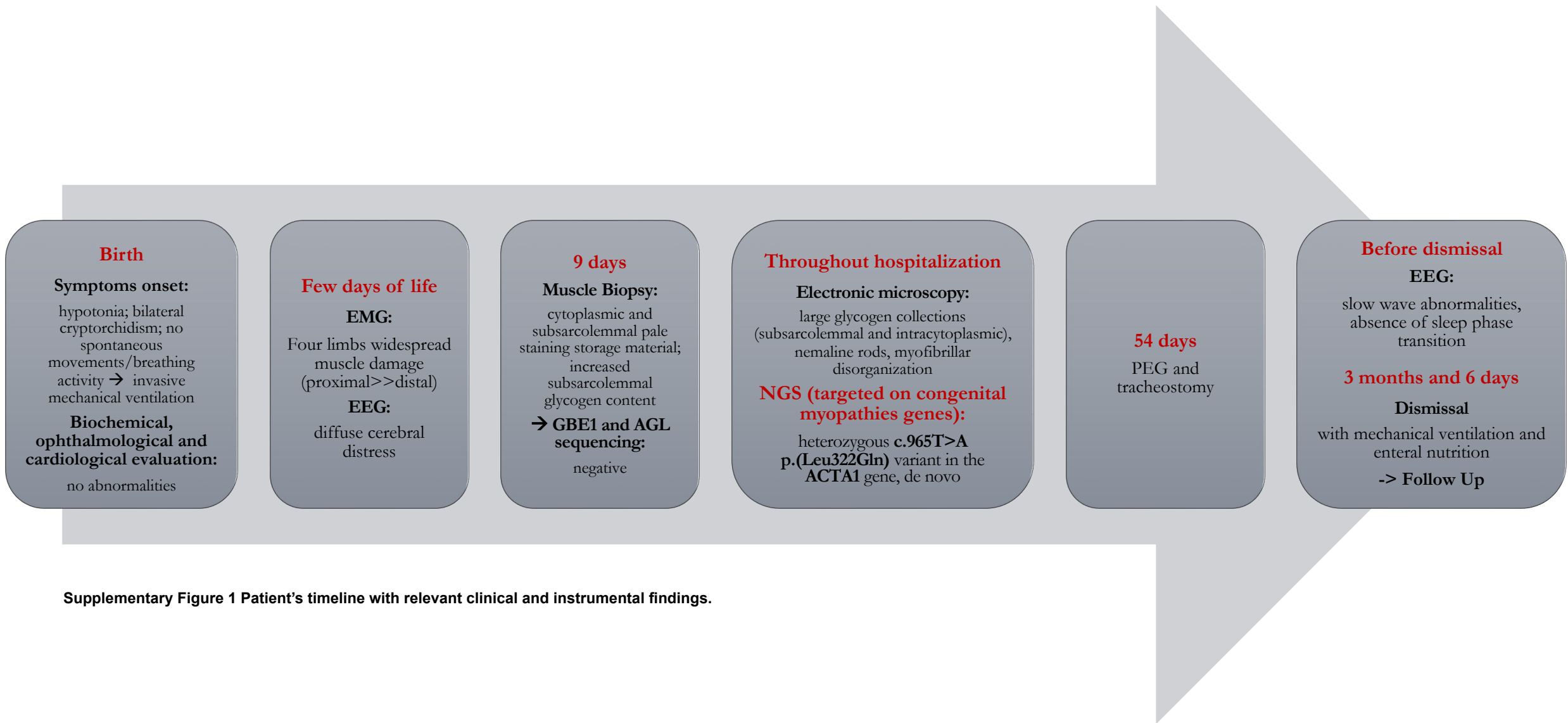

Supplementary Figure 1 Patient's timeline with relevant clinical and instrumental findings.

Supplement: Supplementary file 1 [file Image_1.pdf]
